# Role of luteinizing hormone urinary levels in the diagnostic and therapeutic management of female central precocious puberty

**DOI:** 10.1186/s13052-023-01506-8

**Published:** 2023-08-21

**Authors:** Ilaria Brambilla, Carmen Guarracino, Carmelo Pistone, Catherine Klersy, Amelia Licari, Gian Luigi Marseglia, Enrico Tondina

**Affiliations:** 1 Pediatric Clinic, Fondazione IRCCS Policlinico San Matteo, p.le C. Golgi 19, Pavia, 27100 Italy; 2 Department of Clinical, Surgical, Diagnostic and Pediatric Sciences, Pediatric Unit, University of Pavia, Pavia, Italy; 3 Department of Epidemiology and Biostatistics, Fondazione IRCCS Policlinico San Matteo, Pavia, Italy

**Keywords:** Central precocious puberty, GnRH test, Luteinizing hormone urinary levels (uLH), Diagnosis, Monitoring

## Abstract

**Background:**

Diagnosing central precocious puberty (CPP) requires an integrated approach based on clinical, biochemical and instrumental data. The diagnostic gold standard is represented by GnRH (gonadotropin-releasing hormone) stimulation test. Some undoubted limitations of this procedure led the international scientific community to look for cheaper and less invasive alternative diagnostic methods, such as luteinizing hormone urinary levels (uLH) measurement. This study aims to define the reliability of urinary LH levels as a biomarker of pubertal development, both concerning the initial diagnostic management and the monitoring of patients with central precocious puberty undergoing therapy with GnRH analogues. Furthermore, the study plans to detect the potential association between LH peak serum (pLH) and urinary LH in patients undergoing diagnostic tests with GnRH and to identify a possible cut-off of uLH that may be suggestive of ensued successful hormonal stimulation.

**Methods:**

The study includes 130 female patients with suspected precocious puberty or in follow-up during suppressive therapy. After the collection of the informed consent, the patients underwent clinical evaluation, auxological assessment, and hormone assays (basal levels of LH, FSH, and oestradiol; GnRH stimulating test in patients with suspected precocious puberty; urinary LH assay on the first-morning urine sample, collected after waking up).

**Results:**

Two uLH cut-off values have been identified: the first of 0.25 UI/L [C.I. 95% 0.23–0.27], able to distinguish between pubertal and pre-pubertal patients, the second of 0.45 UI/L [C.I. 95% 0,20 − 0,70] suggestive of occurred hormonal stimulation in patients with diagnosis of CPP at GnRH test. All 30 patients with CPP in follow-up during suppressive therapy presented uLH values ≤ 0.45 IU/L (p^U^ < 0.05), and uLH collected in prepubertal group control.

**Conclusions:**

uLH assays on the first morning urine specimen could be considered a low-cost and minimally invasive tool for precocious puberty diagnosing and monitoring, making possible to be easily performed even by a general pediatrician. Thus, this could help referring only selected patients to pediatric endocrinologists. After an appropriate validation, this approach could reasonably reduce hospital attendance and costs of performing more invasive procedures, with a more significant emotional impact on the pediatric patient.

## Background

The diagnostic approach to the patient with suspected precocious puberty is based on the integrated evaluation of clinical, biochemical, and instrumental data [1]. No single biomarker has yet been identified to discriminate between pubertal and prepubertal subjects [2].

In central precocious puberty (CPP), the activation of the hypothalamic-pituitary-gonadal neuro-endocrine axis has to be detected by a stimulation test with GnRH (gonadotropin-releasing hormone), the diagnostic gold standard [3]. This analysis allows to determine pituitary gonadotropins serum levels (LH and FSH) after appropriate pharmacological stimulation; in particular, the peak value reached by LH after stimulation (pLH > 5 IU/L) and the ratio between the peak of LH and peak of FSH (pLH/pFSH > 1), more than the basal serum concentration of LH (sLH), are the most useful parameters for diagnosis [4]. However, the GnRH test is not free of procedural limitations: the need for venous samples at different times, significant stress for the pediatric patients, procedure costs, the constant monitoring by specialized nursing staff, the risk of intravenous drug administration, and the requirement to remain in a hospital setting for at least 4–5 h, are only a few of them.

Due to these limitations, the international scientific community has been looking for alternative diagnostic methods for several years, and luteinizing hormone urinary levels (uLH) measurement is one of the most appealing [5–7]. ULH is, in fact, less invasive, less costly, and less burdensome for the patient and the healthcare unit.

LH and FSH are secreted in a pulsatile pattern under GnRH stimulation. During puberty, the frequency and amplitude of this secretion increase progressively. In the early stages of pubertal development, the amplification of the secretory pattern of these hormones, particularly the LH’s one, can be observed mainly during the night and then becomes evident during the daytime. As a result, in both sexes, the differential between night-time and daytime secretion peaks of luteinizing hormone (pLH) is clearer in Tanner stages 2 and 3, then becoming progressively less evident with the progression and the end of pubertal development [8, 9].

This study aims to assess the feasibility of using urinary LH, assayed on the first-morning urine, as a biomarker of pubertal development, both in the initial diagnostic approach and the monitoring of patients with central precocious puberty undergoing therapy with GnRH analogues (GnRHa).

## Methods

### Aim, design and setting of the study

This study aims to identify a possible diagnostic role of LH urinary assay in female patients with a clinical suspicion of precocious puberty, as well as evaluate its possible use as a biomarker of therapeutic goal in patients treated with GnRHa.

Two groups of patients were studied: prepubertal subjects (healthy controls) and girls with clinical suspicion of precocious puberty; the second included patients already being treated with GnRHa for a diagnosis of true precocious puberty.

Currently, international guidelines consider the GnRH stimulation test the diagnostic gold standard for central precocious puberty, with a serum LH peak cut-off (pLH) of 5 IU/L [3, 10].

Basal serum LH levels (sLH) are less validated, even though values above 0.3 IU/L can be considered predictive of hypothalamic-pituitary-gonadal axis activation. On the contrary, under 0.2 IU/L sLH values predict no clinical progression toward puberty [3, 10].

The primary aim of the study, in the first group of patients, is to find a possible urinary LH cut-off capable of discriminating between pubertal and prepubertal patients. Those subjects enrolled with anamnestic, clinical, biochemical, and instrumental features of hypothalamic-pituitary-ovarian axis activation were considered pubertal.

Moreover, the study aims to detect the potential association between LH peak serum and urinary LH in patients undergoing diagnostic tests with GnRH and identify a possible cut-off of uLH that may, on its own, be suggestive of ensued successful hormonal stimulation.

In the second group of patients, this study also aims to demonstrate the potential role of uLH as a therapeutic monitoring tool for true precocious puberty. The aim is to establish if the mean uLH values in this group are comparable to the uLH values in the group of controls, confirming a good hormone suppression ensured by the therapy.

### Study subjects

The retrospective monocentric study analyzed clinical, laboratory, and instrumental data of 130 female patients referred to the Outpatient Clinic for Auxology, Diabetology, Endocrinology and Pediatric Gynaecology of the I.R.C.C.S. Policlinico San Matteo in Pavia. The subjects were selected between September 2020 and September 2021, considering those with suspected precocious puberty or in follow-up during suppressive therapy with GnRHa.

After the collection of informed consent by their parents, the patients underwent the following: (i) clinical evaluation, including assessment of the pubertal stage according to Tanner staging; (ii) auxological assessment: height and weight measurement, calculation of body mass index, growth rate, and genetic target; all data were subsequently compared with reference curves for sex, age, and ethnicity; (iii) hormone assays, including basal levels of LH (sLH), FSH (sFSH), and oestradiol (E2), possibly followed, in patients with suspected precocious puberty, by GnRH stimulating test; (iv) urinary LH assay on the first morning urine sample (uLH), collected after waking up (first voided specimen). In particular, the collection of first morning urine samples took place between 08:00 a.m. and 12:00 a.m. to better reflect the nocturnal peak of LH secretion [11, 12]. All patients were asked to urinate, last time before sample collection, no later than 12:00 p.m. of the previous day [11]. The exclusion criteria were male sex, gonadotropin-independent precocious puberty, precocious puberty secondary to endocrinopathies or other organ diseases, estrogen-progestin therapy, night-time enuresis, age under 6 years and not conforming urine sample collection.

The first study group included 100 female subjects aged between 6 and 10 years. Of these, at clinical evaluation 60 girls (age 6.0–10.00) were considered pubertal, with subsequent diagnosis of central precocious puberty, pubertal advancement, or physiological puberty as defined by the Italian Society of Pediatric Endocrinology and Diabetology (SIEDP) guidelines [3]. All patients with suspected precocious puberty who followed the standard diagnostic procedure for CPP were asked to urinate before bedtime and to abstain from fluid consumption until urine collection the following morning (first voided specimen). In the 51 patients who were candidates for GnRH testing, when CPP was suspected, the urine sample was collected before performing the procedure, to avoid any influence on uLH concentrations. The remaining 40 girls (age 6.0–9.0) of the first study group were considered prepubertal at clinical evaluation and thus included in the control population.

The second study group included 30 patients (age 6.00–11.92) diagnosed with central precocious puberty confirmed by GnRH test (pLH > 5 IU/L). All enrolled patients received suppressive therapy with GnRHa (triptorelin) for at least 6 months. The instructions given to these patients for urine sampling were the same as in the first group: collection occurred in the morning, immediately after waking up (between 8:00 and 12:00 a.m.), and before further biochemical and/or instrumental investigations were carried out. No patients were excluded from the study because of inadequate urine specimen collection.

### Hormonal assays

All patients not in the control group underwent venous blood sampling for basal pituitary gonadotropin (LH and FSH) assays. Following the diagnostic procedure recognized by the SIEDP [3] in suspected central precocious puberty, GnRH stimulation tests were performed on 51 of them: after intravenous administration of a bolus of GnRH (Relefact®), amounting to 100 µg/m², serum FSH and LH levels were measured at 0, 15, 30, 60 and 90 min. LH and FSH concentrations were measured by chemiluminescence (IMMULITE 2000) and expressed in IU/L.

Urinary LH was assayed on the first-morning urine sample (first voided specimen) to assess the night-time secretory peak of luteinizing hormone and subsequently corrected for the subject’s urinary creatinine. Urines were stored without preservative materials at 4 °C until the assays were carried out. All tests were performed by the end of the collection day. In particular, the samples were not frozen in order to avoid the physical degradation of LH subunits [13]. The urinary LH from all urine samples was quantified by IMMULITE 2000, an automated immunoassay system (Siemens Healthcare Diagnostics Products Ltd., Los Angeles, USA). The IMMULITE system is a solid-phase, two-site chemiluminescent immunometric assay; the method specifically detects the β-subunit of the hormone both in the intact and free subunit. Urine samples were assayed after centrifugation at 3000 rpm for 5 min at room temperature. Urinary LH concentrations were expressed in IU/L. The analytical sensitivity of the method is equal to 0.05 mUI/ml, and its coefficient of variation (cv) is less than 10%, inversely proportional to the concentration of detectable uLH on the urinary sample.

### Statistical analysis

Descriptive statistical analysis was performed for all variables. Categorical variances were given as absolute values and percentages, while continuous variables were defined as means ± standard deviation (SD). The processing of the collected data was carried out using STATA statistical software (version 16.1).

For the definition of the *cut-off* value of urinary LH, the area under the curve (AUC) ROC was calculated with a corresponding confidence interval [C.I. 95%]. With 100 patients and a ratio of pre-pubertal to pubertal subjects of 0.67, it was possible to elicit as significant a difference between an area under the ROC curve of 0.95 (excellent) and an area of 0.85 (very good) with a type I error of 5% and a power of 90%.

A statistically significant association between the parameters under investigation was established through the *Student’s* t-test for the comparison of symmetrically distributed parametric means, while the Mann-Whitney U-test was used for asymmetric variables.

In contrast, Fisher’s exact test (or *chi*² exact test) was used to test hypotheses involving two dichotomous nominal variables on small samples.

All analyses were conducted considering a *p-value* of less than 0.05 to be statistically significant.

## Results

The 100 female patients in the first study group had an average age of 7.79 years ± 1.06. Of these, 40 patients were considered prepubertal (40%) with an average age of 7.35 years ± 1.04, while 60 patients were pubertal (60%) with an average age of 8.08 years ± 0.98.

According to the Tanner clinical staging, patients were also distributed into four distinct subclasses: 36% of subjects with Tanner stage 1, 42% with Tanner stage 2, 21% with Tanner stage 3, and only 1% with Tanner stage 4. Patients with complete pubertal development, Tanner stage 5, were not assessed. Specifically, all 60 pubertal patients presented thelarche (Tanner stage B2 or higher); among the 40 prepubertal patients, only 17 (42.5%) showed mammary granulation. On non-parametric analysis, according to Fisher, this distribution was statistically significant (p < 0.05).

The basal serum LH concentration (sLH) in the 95 patients who had venous sampling averaged 0.48 IU/L ± 0.33; considering the two subgroups, the mean sLH was 0.14 IU/L ± 0.05 in prepubertal patients and 0.68 IU/L ± 0.46 in pubertal patients (p < 0.05).

**Fig. 1:**
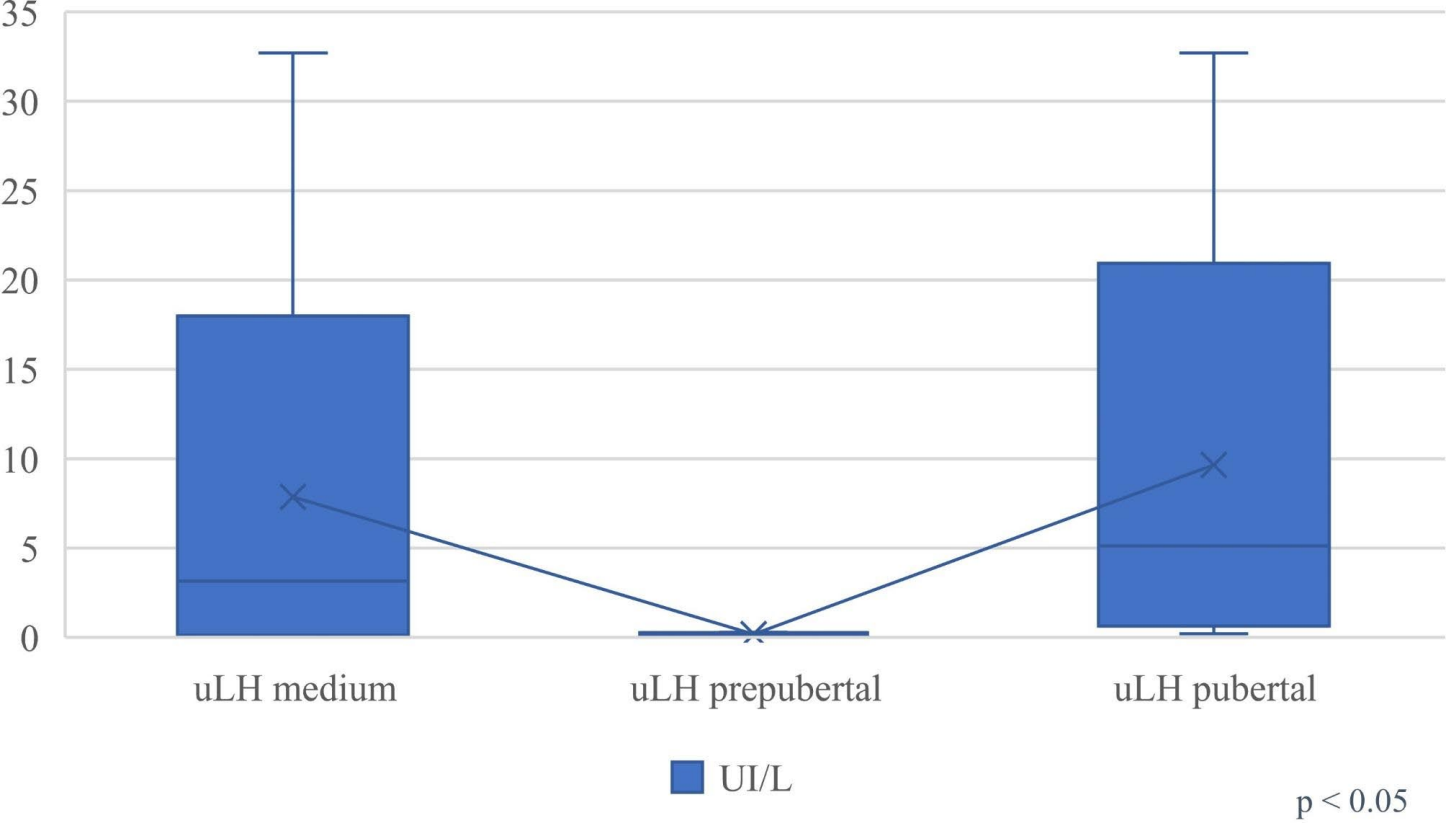
Urinary LH concentrations (uLH) in the first study group

ULH levels, considering all the enrolled patients, averaged 3.14 IU/L ± 3.00, with a respective distribution of: 0.16 IU/L ± 0.05 in prepubertal patients and 5.12 IU/L ± 4.05 in pubertal patients (p < 0.05) (Fig. 1).

Finally, in the 51 patients (74.5% pubertal and 25.5% prepubertal) who underwent the GnRH test for the diagnosis of central precocious puberty, the mean peak LH (pLH) reached in the prepubertal group after pharmacological stimulation was 3.2 IU/L ± 1.02 (≤ 5 IU/L), while for the pubertal group it was 14.05 IU/L ± 12.85 (> 5 IU/L) (p < 0.05).

When analyzing the raw data on the GnRH test results, it was found that 76.5% of the patients with pLH equal to or less than 5 IU/L were considered to be prepubertal, whereas almost all of the patients considered to be pubertal had a pLH greater than 5 IU/L (the GnRH test gave a negative result in only 10.5% of the pubertal patients, i.e., pLH ≤ 5 IU/L) (p < 0.05). (Table 1)

**Table 1:** Characteristics of the female patients in the first group of study

	N. FEMALE PATIENTS	MEAN AGE (years)	sLH (UI/L)	pLH (UI/L)	uLH (UI/L)
**PREPUBERAL** **TANNER < 2**	40	7,35 ± 1,04	0,14 ± 0,05	3,2 ± 1,02	0,16 ± 0,05
**PUBERAL** **TANNER ≥ 2**	60	8,08 ± 0,98	0,68 ± 0,46	14,05 ± 12,85	5,12 ± 4,05
**TOTAL**	100	7,79 ± 1,06	0,48 ± 0,33	-	3,14 ± 3,00

**Fig. 2:**
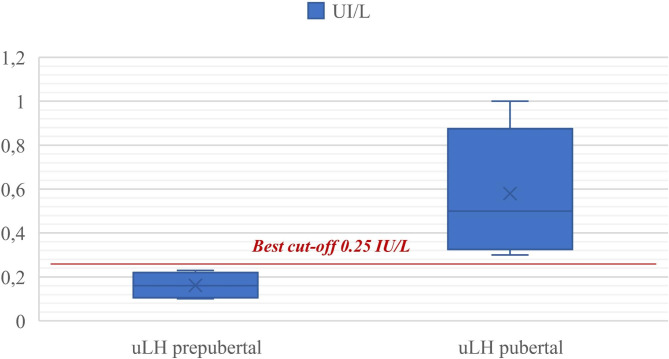
uLH best cut-off for determination of pubertal development status

Concerning the primary objective of the study, after ROC curve analysis of the urinary LH values collected from the entire population (100 patients, of which 40 prepubertal and 60 pubertal), the uLH value = 0.25 IU/L [C.I. 95% 0.23–0.27] was identified as the best cut-off for discriminating the actual onset of puberty (Fig. 2). According to the classification proposed by Swets [14], this value is highly accurate: the AUC (Area Under Curve) is 0.98 [0.9 < AUC < 1.0], resulting in high sensitivity (Sn) and specificity (Sp) of the test (98% and 97% respectively). The positive predictive value (PPV) and negative predictive value (NPV) were 98.3% and 97.5%.

**Fig. 3:**
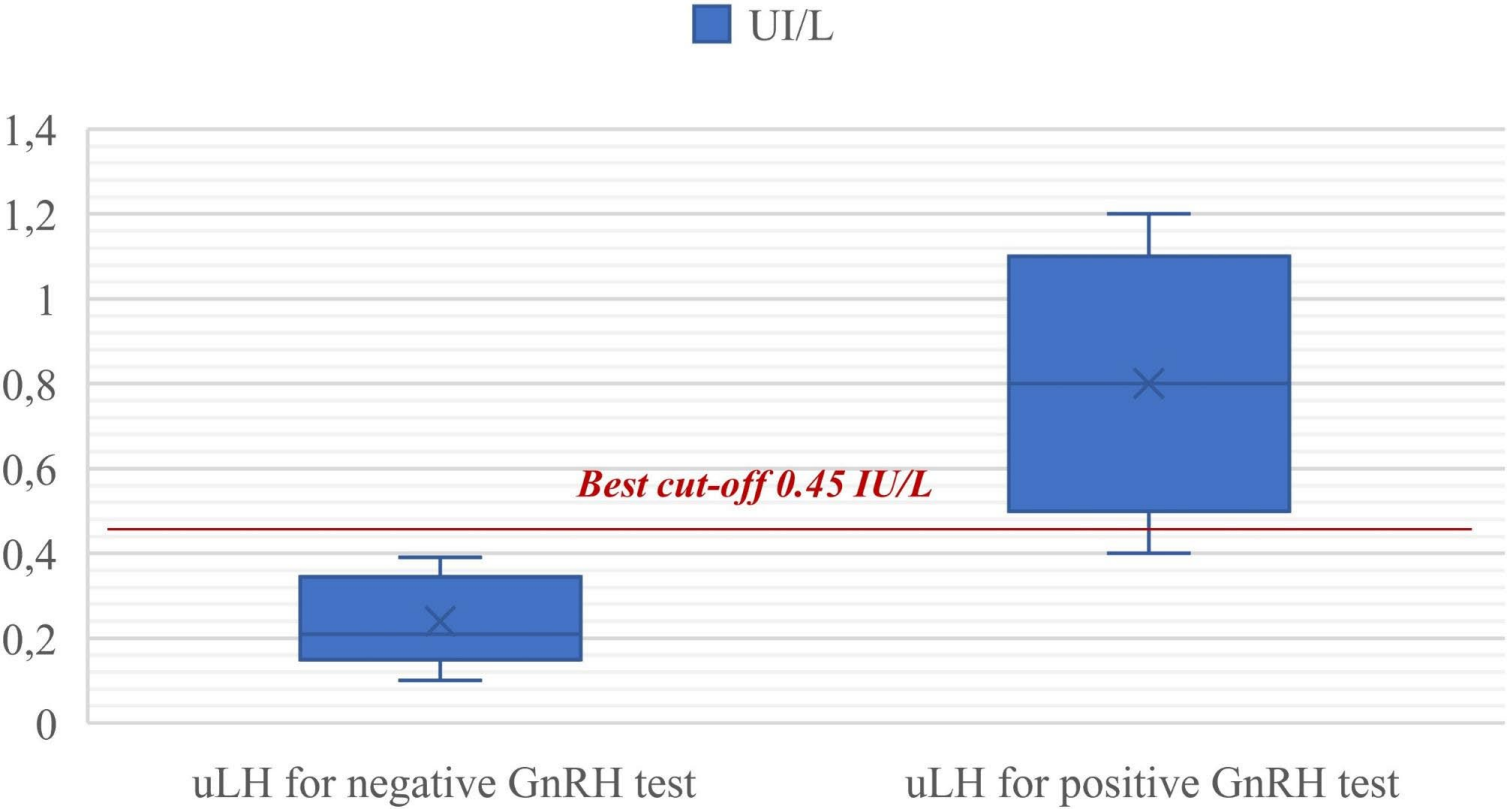
uLH best cut-off for determination of the response to the GnRH-test.

Regarding the sole GnRH-tested patients (51 in total, of which 13 were prepubertal and 38 pubertal), ROC curve analysis of urinary LH values (collected before the test was performed) identified uLH = 0.45 IU/L [C.I. 95% 0.20–0.70] as the *best cut-off* for hormonal stimulation (Fig. 3). With an AUC of 0.99, this test is highly accurate for the Swets [14] classification [0.9 < AUC < 1.0]; however, while the test has an excellent specificity (Sp = 99%) and a good positive predictive value (PPV = 99%), its sensitivity and negative predictive value are not as good (Sn = 77% and NPV = 74.1%).

In Table 2 is showed the distribution of patients undergoing GnRH test, with respect to uLH value and pLH value after stimulation. In Table 3 is showed predictive power of uLH for discrimination between pubertal/prepubertal patients and for identification of occurred hormonal stimulation.

**Table 2:** Distribution of patients undergoing GnRH testing with respect to uLH value and peak serum LH after stimulation

pLH at TEST	uLH ≤ 0,45 UI/L	uLH > 0,45 UI/L	Total
**≤ 5 UI/L**	17	0	17
**> 5 UI/L**	1 (uLH = 0,4)	33	34
**Total**	18	33	51

**Table 3:** Predictive power of urinary LH levels (uLH) for discrimination between pubertal/prepubertal patients (1) and for identification of occurred hormonal stimulation (2)

Best *cut-off* uLH (1)	IC 95%	Sn	Sp	VPP	VPN	AUC
**0.25** UI/L	[0,23 − 0,27]	98%	97%	98,3%	97,5%	0.98
**Best c** ***ut-off*** **uLH peak (2)**	**IC 95%**	**Sn**	**Sp**	**VPP**	**VPN**	**AUC**
**0,45** UI/L	[0,20 − 0,70]	77%	99%	99%	74,1%	0,99

The second study group included a total of 30 female patients with a mean age of 9.00 ± 1.45 years. All subjects underwent stimulation testing with GnRH and were diagnosed with true precocious puberty (pLH > 5 IU/L). Assumed the rapid progression of the clinical developmental in these girls, suppressive therapy with GnRHa (triptorelin) was started for all of them.

At the beginning of the treatment, according to Tanner’s staging, 20 patients (66.6%) had stage 2; 9 patients (30%) had stage 3; 1 patient (3.4%) was in stage 4. None of the patients was assigned to a Tanner stage 1 (prepubertal) or 5 (fully developed).

**Fig. 4:**
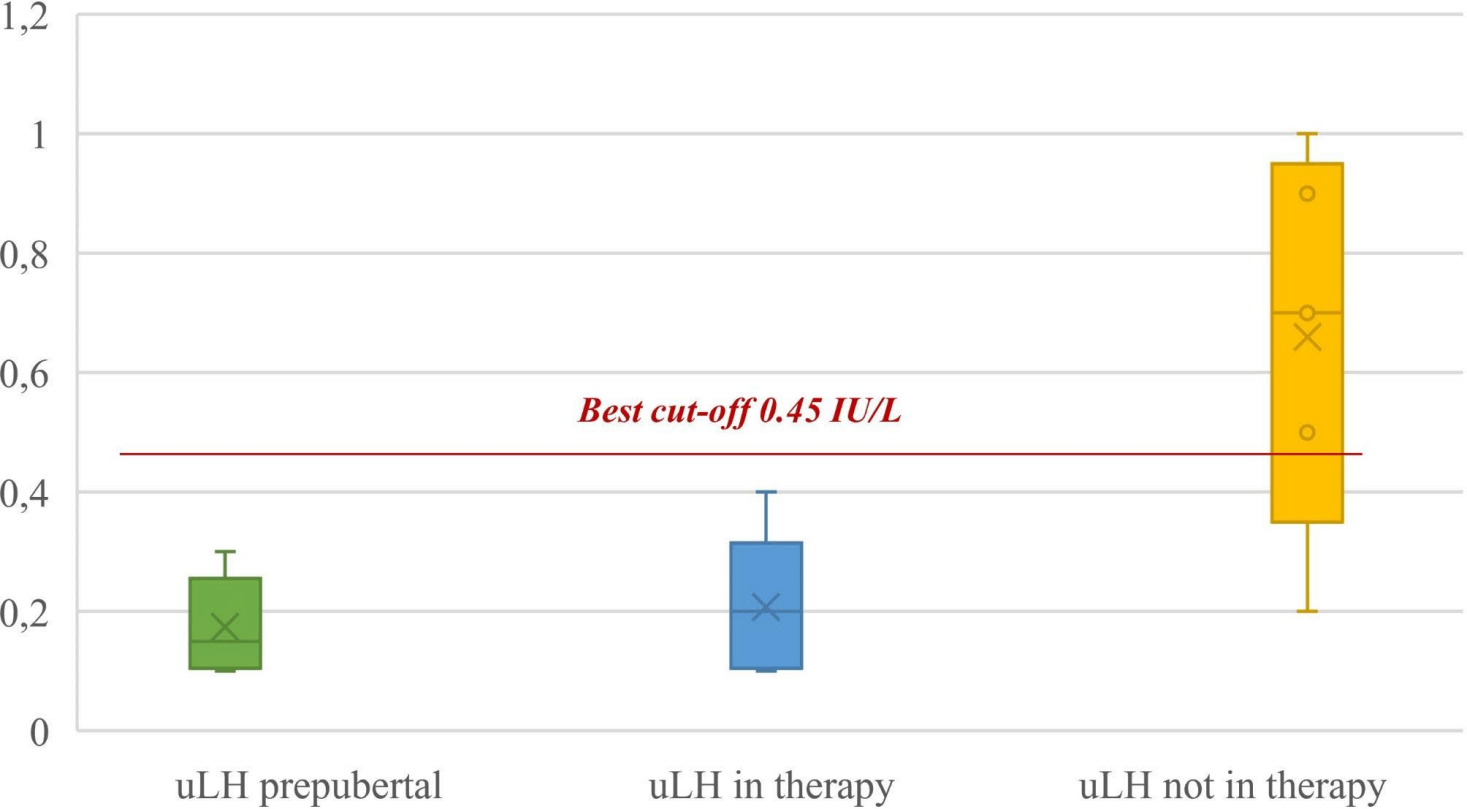
Comparison of urinary LH values (IU/L) in the main population groups

Regarding urinary LH levels, this second group of patients showed uLH mean values of 0.20 IU/L ± 0.09: in particular, 22 patients (73.3%) had uLH values ≤ 0.25 IU/L and only 8 patients (26.7%) had values above this *cut-off*. All 30 patients on GnRHa therapy presented uLH values ≤ 0.45 IU/L (Fig. 4). These results appear to be statistically significant (p < 0.05) and similar to the prepubertal population control group.

In Table 4 is summarised the comparison between uLH values in the main population groups of study.

**Table 4:** Comparison of urinary LH values in the main population groups

	n.	Mean	SD	min	p50	max	% Subjects uLH ≤ 0,45 UI/L
**uLH in prepubertal patients**	40	0,16 UI/L	0,05	0,10	0,20	0,30	100%
**uLH in pubertal patients treated with GnRHa**	**30**	**0,20 UI/L**	**0,09**	**0,10**	**0,20**	**0,40**	**100%**
**uLH in pubertal patients NOT treated with GnRHa**	60	5,12 UI/L	4,05	0,20	2,10	32,7	23,3%

**AUC** area under the curve. **C.I.** confidence interval. **GnRHa** GnRH analogs. **NPV** negative predictive value. **pLH** luteinizing hormone peak serum. **p50** median.**PPV** positive predictive value. **SD** standard deviation. **Sn** sensitivity. **Sp** specificity. **sLH** luteinizing hormone basal serum. **uLH** luteinizing hormone urinary levels

## Discussion

In accordance with previously published clinical studies [13, 15–17], the uLH cut-off values we identified in pubertal and prepubertal patients are well correlated with baseline LH (sLH) and its peak after stimulation (pLH). Compared to the study by Lucaccioni et al. [18], our cut-off values also have greater statistical sensitivity and specificity in predicting precocious puberty, probably due to greater sample size and a major homogeneity of the examined sample.

Integrated clinical, biochemical, and instrumental data provided by the 100 patients examined in the first study group allowed us to identify a uLH value capable of discriminating between pubertal and prepubertal patients accurately: in particular, the cut-off of 0.25 IU/L, not only has a high positive and negative predictive value but owns a good narrow confidence interval [C.I. 95%: 0.23–0.27] too. This result is further supported by what is daily observed in our clinical experience diagnosing central precocious puberty: in this study case series, urinary concentrations of luteinizing hormone above 0.25 IU/L are associated with clinical, biochemical, and radiological findings compatible with effective activation of the hypothalamic-pituitary-ovarian axis.

The study was able to document a statistically significant discriminating power of urinary LH regarding the pLH increment for patients who underwent stimulating tests with GnRH. The cut-off we identified, hypothetically able to independently predict the dynamic test response, is 0.45 IU/L (ROC test with an AUC of 0.99).

Moreover, the reliability of the obtained best cut-off is supported by using a standardized test for sLH measurement: uLH assay is carried out by translating the serum LH test into the urinary matrix. As underlined by Bourguignon et al. [12], a possible bias in the validation of the uLH values could be the collection and the first morning urine conservation procedures. Regarding the timing, in agreement with Kolby et al. [11], patients and their families were recommended to collect urine samples at the most representative time of LH nocturnal peak (between 08:00 a.m. and 12:00 a.m.). They were also told not to urinate after 12:00 p.m. of the day before.

To confirm the importance of an adequate urine collection procedure, it should also be remembered that the circadian rhythm of sLH, and consequently of uLH, is particularly marked in prepubertal girls up to Tanner stage 3 [12].

Although home urinary samples could underlie some limitations concerning the correct collection procedure, as reported by Xu et al. [19], it represents an undoubtedly valid tool as a non-invasive method to diagnose CPP. Concerning the storage of urinary samples, the choice not to freeze but to store urines at a controlled temperature of 4 °C derives from the need to ensure greater stability of the LH molecule, in accordance with what was established by Demir et al.[13].

This encouraging statistical result contributes to the proposed validation of uLH as a routine diagnostic procedure to distinguish between prepubertal and pubertal subjects, and it is associated with a relatively wide confidence interval [C.I. 95%: 0.20–0.70], which minimally affects the predictive sensitivity of the test under investigation. This analytical issue could be relieved by expanding the sample size case series or drawing a similar study on a homogeneous but independent population, possibly involving other qualified centres.

In a clinical context, this statistical aspect translates into greater difficulty interpreting the few uLH values between 0.25 and 0.45 IU/L, needing integration with clinical and instrumental data. While values of uLH less than or equal to 0.25 IU/L indicate a prepubertal stadium, and values greater than 0.45 IU/L predict activation of the hypothalamic-pituitary-gonadal axis (pLH greater than 5 IU/L), values in the above range, taken individually, cannot be related immediately to a specific group of patients and must be appropriately contextualized.

In the second group of patients, consisting of female subjects suffering from rapidly progressive precocious puberty, treated with GnRH analogues (triptorelin), the study shows that uLH can be used to monitor therapeutic efficacy. In this case, the average uLH values found in patients undergoing treatment were identical to those reported for the control group involving prepubertal patients.

Moreover, this result appears to have a high statistical significance, especially compared to the average uLH values recorded in pubertal patients not treated with GnRH analogues. According to previous data published by Zhan, Zhung and Tripathy [5, 20, 21], also in this study group, uLH levels significantly decreased after six months of treatment with GnRHa. The explanation of this result lies in the mechanism of action of the therapy itself: GnRH analogues exert a suppressive action at the level of the central nervous system, effectively interrupting the pulsatile secretion of pituitary gonadotropins (LH and FSH), with a consequent reduction in their serum and urinary concentrations [22].

One of the undoubtedly most promising data relates to the percentage achieved (100%) of prepubertal and pubertal patients on triptorelin therapy who present urinary LH values below the cut-point of 0.45 IU/L, as a result of actual functional inactivity of the endocrinological hypothalamic-pituitary-ovarian axis.

In a broader perspective, urinary LH values could thus be considered valid biomarkers of clinical response to suppressive therapy with GnRH analogues.

Potential study limitations could be some procedural problems inherent in the urine collection and storage method and the lack of a confirmatory study on a homogeneous subject sample.

## Conclusions

The choice of topic for this study was undoubtedly influenced by the particular interest aroused among those involved in pediatric endocrinology about the increased incidence of precocious puberty during the SARS-COV2 pandemic: the forced sedentary lifestyle imposed by lockdown periods has forced many children to abstain from physical activity, habitually consume harmful comfort food and spend many hours in front of bright screens, factors that seem to determine early pubertal onset [23–26].

The limitations provided by the lockdown, including the restrictions on access to specialist public health clinics for non-urgent problems, allow the possibility of seeking alternative diagnostic strategies, such as urinary dosing of luteinizing hormone to diagnose and monitor central precocious puberty. According to the most up-to-date recommendations, the GnRH stimulation test remains the gold standard for identifying the hypothalamic-pituitary-gonadal axis activation; this test is, however, characterized not only by a certain degree of invasiveness and high costs but also by the apparent need to keep the patient in the hospital for several hours, an event that should be reduced to what is strictly necessary, especially during a pandemic period like this.

The primary objective of this study is to validate a low-cost, minimally invasive method for precocious puberty diagnosing and monitoring that can be rapidly and easily performed, even outside hospitals, such as uLH assays on the first voided specimen of the morning urine (taking care to illustrate to the parent the correct way to collect the sample).

Referring to urinary LH levels only, even in a territorial setting, a general pediatrician could select patients with suspected precocious puberty to send to an endocrinological third-level centre. After an appropriate validation by the most authoritative scientific societies of pediatric endocrinology, this approach could reasonably reduce hospital attendance and the costs of performing more invasive procedures with a more significant emotional impact on the pediatric patient.

Regarding the need to validate the obtained results, an essential path to attribute a globally recognized diagnostic role to urinary LH, it is important to point out that despite the high statistical accuracy of the best cut-off obtained on this population, an expansion of the sample size is still necessary to reduce the range of interpretative uncertainty concerning values between 0.25 IU/L and 0.45 IU/L as much as possible.

Although the high sensitivity and specificity of the cut-point of 0.25 IU/L to identify pubertal patients in a mixed population (pubertal and prepubertal), as well as the high power of this assay in predicting serum pLH greater than 5 IU/L in the GnRH test for urinary LH values greater than 0.45 IU/L, it remains challenging to define pubertal patients who are in an intermediate condition between these two biochemical realities.

Finally, about the possibility of using the urine-based luteinizing hormone test for the therapeutic monitoring of drug-treated precocious puberty patients, this study further highlights the usefulness of observing the hormone suppression brought by using GnRH analogues in a non-invasive way.

In particular, in assessing therapeutic effectiveness, the role of urinary LH even seems to exceed its power as a therapeutic and diagnostic predictor. In pubertal patients examined at least six months after the start of triptorelin therapy, urinary hormone levels are not only significantly similar to those of prepubertal patients. However, they are also stably below the cut-off of 0.45 IU/L, indirectly suggesting complete suppression of the hypothalamic-pituitary-gonadal axis. The need to investigate this research topic through further studies, once its usefulness in clinical practice has been recognized, becomes even more urgent given the scarce availability of relevant studies in scientific literature.

## Data Availability

The datasets used and/or analysed during the current study are available from the corresponding author upon reasonable request.

## Abbreviations

AUC: area under the curve
CPP: central precocious puberty
E2: oestradiol
FSH: follicle-stimulating hormone
GnRHa: GnRH analogs
GnRH: gonadotropin-releasing hormone
LH: luteinizing hormone
pFSH: follicle-stimulating hormone peak serum
pLH: luteinizing hormone peak serum
SD: standard deviation
sFSH: follicle-stimulating hormone basal serum
sLH: luteinizing hormone basal serum
uLH: luteinizing hormone urinary levels
